# Plasma Figure Correction Method Based on Multiple Distributed Material Removal Functions

**DOI:** 10.3390/mi14061193

**Published:** 2023-06-03

**Authors:** Xiang Wu, Bin Fan, Qiang Xin, Qian Luo, Junming Shao, Guohan Gao, Peiqi Jiao

**Affiliations:** 1 National Key Laboratory of Optical Field Manipulation Science and Technology, Chinese Academy of Sciences, Chengdu 610209, China; 2 Institute of Optics and Electronics, Chinese Academy of Sciences, Chengdu 610209, China; 3 University of Chinese Academy of Sciences, Beijing 100049, China

**Keywords:** reactive ion etching, figure correction, material removal distribution, material removal functions

## Abstract

In the process of plasma figure correction for a quartz sub-mirror, the plasma parallel removal process and ink masking layer are combined for the first time. A universal plasma figure correction method based on multiple distributed material removal functions is demonstrated, and its technological characteristics are analyzed. Through this method, the processing time is independent of the workpiece aperture, which saves time for the material removal function to scan along the trajectory. After seven iterations, the form error of the quartz element is converged from the initial figure error of ~114 nm RMS to a figure error of ~28 nm RMS, which shows the practical potential of the plasma figure correction method based on multiple distributed material removal functions in optical element manufacturing and the possibility of becoming a new stage process in the optical manufacturing chain.

## 1. Introduction

Ultra-precision optical components are increasingly used in astronomical observation, aerospace, earth imaging, and many other fields. Typically, in order to improve material removal efficiency and full-band processing accuracy, the original substrate of an optical component needs to be figured and ultra-precisely polished through a series of surface processing techniques. The ultra-precision optical manufacturing chain mainly includes milling, grinding, and subsequent polishing processes, such as bonnet polishing (BP) [1,2,3], magneto-rheological finishing (MRF) [4], and ion beam figuring (IBF) [5]. These material processing methods can improve the surface quality and accuracy of components. In addition, for optical components with specific requirements, such as the formation of smooth, defect-free surfaces for fused silica optics, emerging laser processing methods [6,7] are used in the manufacturing chain. The current mainstream optical precision machining technologies, such as bonnet polishing, magneto-rheological polishing, robot automatic polishing [8], and plasma jet polishing [9], were developed mainly based on computer-controlled optical surfacing (CCOS) proposed by Itek Inc [10]. In CCOS, the tool is usually traversed along a regular path over the entire surface to remove the material or to improve the medium frequency error by increasing the randomness of the path [11,12,13]. These methods are essentially based on a removal mode where the material removal functions (MRFs) are scanned point by point along a predetermined trajectory to achieve figure correction. It is clear that an optical processing method with high material removal efficiency and low surface damage in the ultra-precision optical manufacturing chain will have the potential to significantly reduce the manufacturing cycle time of optical components.

Plasma jets with temperatures that can reach thousands of degrees Kelvin [9] and the thermal plasma generated at high temperature and high pressure can easily cause thermal damage to material surfaces. Reactive ion etching (RIE), as part of the process in IC processing, uses low-temperature plasma as a tool in a low-pressure environment to transfer the pattern on the mask plate to the substrate with high processing accuracy, anisotropy, and etching selectivity [14,15,16,17]. In the field of semiconductor manufacturing, this plasma processing method can even achieve atomic-level processing accuracy. The discharge mechanism and the processing characteristics of dry etching have been reported in many studies [18,19,20], including the mechanism of electron heating in plasma [21], the temporal and spatial evolution of electron heating in different modes [22], and the optimization of etching uniformity of plasma [23,24]. In our previous research, we used this reactive ion etching technology with low temperature, high precision, and non-contact processing characteristics to process large-aperture ultra-light polyimide film substrate. A 400 mm aperture membrane substrate was figure-corrected by reactive ion etching from the initial figure error of 105 nm rms to the final figure error of ~17 nm rms [25]. This makes it possible to fabricate high-precision optical substrates of ultra-lightweight diffractive lenses. In addition, in reactive ion etching, we obtained the tailored material removal distribution on polyimide membrane by introducing additional electrodes to tune the plasma sheath properties. This provides an effective and universal solution for achieving customized material removal distribution in the full-aperture range, as well as for improving the uniformity of reactive ion etching [26].

However, automated fabrication of the masking layer during plasma figure correction has not yet been implemented. The use of manual cutting or lithography to obtain the masking layer is tedious and time-consuming, which greatly reduces the efficiency of plasma figure correction. Moreover, the role played by reactive ion etching processing in the optical manufacturing process chain has not yet been defined, and requires further research on the characteristics of plasma figure correction. Different from the usual processing model where MRF is scanned point by point along a predetermined trajectory to achieve material removal, RIE is capable of simultaneously etching the sample surface in the full-aperture range. Obviously, the larger the aperture of the sample, the more time will be saved in material processing. In this paper, a plasma figure correction method based on multiple distributed material removal functions is proposed based on the high efficiency of parallel processing and low surface damage of RIE. Due to its excellent characteristics of rapid convergence of form error, this plasma figure correction method is mainly used in the transition stage between the early milling process and the late ultra-precision polishing. In the etching process, the UV-cured ink masking layers were used to divide and isolate the etched area and the non-etched area, and material removal was performed by multiple distributed material removal functions for areas with sufficiently large form error. The plasma processing mode based on parallel removal is expected to become an efficient and high-precision processing procedure in the manufacturing method of optical elements.

## 2. Theory and Mathematical Model

**Figure 1:**
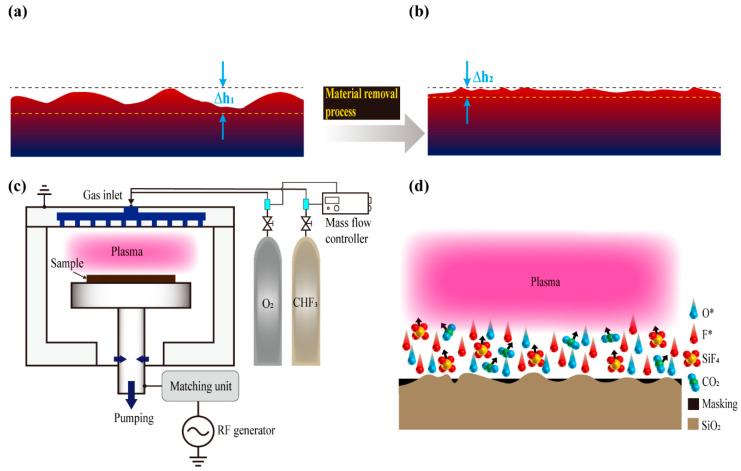
Experimental setups and plasma figure correction process: (**a**) schematic diagram of pending plasma figure correction stage; (**b**) schematic diagram of pending ultra-precision machining stage; (**c**) schematic diagram of the experimental setups; (**d**) schematic diagram of the material removal process.

As shown in Figure 1a, the optical sample usually has a large form error after being milled or roughly polished, and a large amount of material needs to be removed from the sample surface in the full-aperture range. In this case, the proposed plasma figure correction method based on multiple distributed material removal functions is used for this processing stage. It enables efficient convergence of initial form errors and provides a transition (as shown in Figure 1b) to subsequent ultra-precision optical machining stages (such as magnetorheological polishing, ion beam figuring, etc.). The proposed plasma figure correction method based on multiple distributed material removal functions for the efficient figure correction of optical components is originated from a capacitively coupled plasma (CCP) etching technology, which is characterized by low temperature and low pressure. Figure correction was performed on the apparatus as shown in Figure 1c. The reaction gas is a mixture of CHF_3_ and O_2_; oxygen as an auxiliary gas is mainly used to reduce the deposition of fluorocarbons on the surface [27]. The substrate is connected to a matcher, which is connected to the RF power supply. The matcher is used to match the impedance between the source and the target to maximize the forward RF power. Under RF excitation, a glow discharge is generated in the flat capacitor, causing a sheath layer to form on the surface of the element placed on a water-cooled platform. At the same time, as shown in Figure 1d, the sheath voltage drives the charged particles in the plasma to move to the workpiece surface, causing them to react chemically with the atoms on the surface and form volatile gas molecules. Finally, this reaction product is pumped away to form material removal.

In the proposed plasma figure correction method, the material removal can be described as:

(1) $$R_{n}\left(x,y\right)=\left[\begin{array}{ccccc} r\left(1,1\right) & r\left(1,2\right) & \cdots & r\left(1,y-1\right) & r\left(1,y\right)\\ r\left(2,1\right) & r\left(2,2\right) & \cdots & r\left(2,y-1\right) & r\left(2,y\right)\\ \vdots & \vdots & \vdots & \vdots & \vdots \\ r\left(x-1,1\right) & r\left(x-1,2\right) & \cdots & r\left(x-1,y-1\right) & r\left(x-1,y\right)\\ r\left(x,1\right) & r\left(x,2\right) & \cdots & r\left(x,y-1\right) & r\left(x,y\right) \end{array}\right]=\sum_{i=1}^{n}t_{i}\cdot MRF_{i}$$

(2) $$MRF_{i}=\left[\begin{array}{ccccc} v_{i}\left(1,1\right)\cdot k_{i}\left(1,1\right) & v_{i}\left(1,2\right)\cdot k_{i}\left(1,2\right) & \cdots & v_{i}\left(1,y-1\right)\cdot k_{i}\left(1,y-1\right) & v_{i}\left(1,y\right)\cdot k_{i}\left(1,y\right)\\ v_{i}\left(2,1\right)\cdot k_{i}\left(2,1\right) & v_{i}\left(2,2\right)\cdot k_{i}\left(1,2\right) & \cdots & v_{i}\left(2,y-1\right)\cdot k_{i}\left(2,y-1\right) & v_{i}\left(2,y\right)\cdot k_{i}\left(2,y\right)\\ \vdots & \vdots & \vdots & \vdots & \vdots \\ v_{i}\left(x-1,1\right)\cdot k_{i}\left(x-1,1\right) & v_{i}\left(x-1,2\right)\cdot k_{i}\left(x-1,2\right) & \cdots & v_{i}\left(x-1,y-1\right)\cdot k_{i}\left(x-1,y-1\right) & v_{i}\left(x-1,y\right)\cdot k_{i}\left(x-1,y\right)\\ v_{i}\left(x,1\right)\cdot k_{i}\left(x,1\right) & v_{i}\left(x,2\right)\cdot k_{i}\left(x,2\right) & \cdots & v_{i}\left(x,y-1\right)\cdot k_{i}\left(x,y-1\right) & v_{i}\left(x,y\right)\cdot k_{i}\left(x,y\right) \end{array}\right]$$

(3) $$K_{MRF}=\left[\begin{array}{ccccc} k_{1}\left(1,g\right) & k_{2}\left(1,g\right) & \cdots & k_{n-1}\left(1,g\right) & k_{n}\left(1,g\right)\\ k_{1}\left(2,g\right) & k_{2}\left(2,g\right) & \cdots & k_{n-1}\left(2,g\right) & k_{n}\left(2,g\right)\\ \vdots & \vdots & \vdots & \vdots & \vdots \\ k_{1}\left(u-1,g\right) & k_{2}\left(u-1,g\right) & \cdots & k_{n-1}\left(u-1,g\right) & k_{n}\left(u-1,g\right)\\ k_{1}\left(u,g\right) & k_{2}\left(u,g\right) & \cdots & k_{n-1}\left(u,g\right) & k_{n}\left(u,g\right) \end{array}\right],\left(u,g\right)\in \left(x,y\right)$$

(4) $$k_{i}\left(u,g\right)=\left\{\begin{array}{l} \begin{array}{ll} 1 & u,g\in MReg_{i}\left(x,y\right) \end{array}\\ \begin{array}{ll} 0 & u,g\in EReg_{i}\left(x,y\right) \end{array} \end{array}\right.$$

where *r*(*x*,*y*) represents the depth of material removal at the point (*x*,*y*); *t_i_* and *MRF_i_* are respectively expressed as the time and the material removal function in the *i*^th^ figuring cycle; *v_i_*(*x*,*y*) is the plasma etching rate. Due to the anisotropy of dry etching [20], in the same etching process, the etching rate of each point on the etched surface can be regarded as almost equal to a certain extent, that is,

(5) $$\Delta =\left[v_{i}\left(u,g\right)-\frac{1}{N}\sum_{u=1}^{x}\sum_{g=1}^{y}v_{i}\left(u,g\right)\right]\approx 0$$

In Equation (2), *k_i_*(*x*,*y*) is the influence factor of *MRF_i_*, which controls each distributed removal function because it determines the shape of the *i*^th^ masking layer. The etched area *EReg_i_*(*x*,*y*) for each figuring cycle and the set of influence factors *K_MRF_* are pre-planned according to the initial from error of the workpiece detected by an interferometer. In CCOS, the residual error of the elements is mainly reduced by point-by-point removal, i.e., MRF is matched with the machining trajectory and combined with the planning of the dwell time to obtain the convergence of the residual error [28,29]. In RIE figuring, we reduced the figure error by parallel removal of the whole of the planned etched area, which could also be considered as a new technology for subtractive manufacturing. In the process, we gradually decreased the large form residuals in the etched area on the workpiece surface by layer by layer etching.

**Figure 2:**
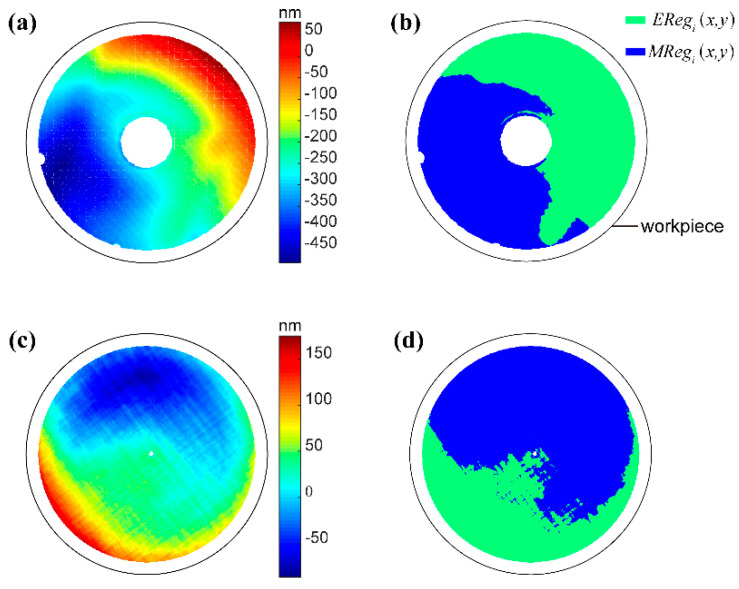
Etched region and masking layer region of the different surface shapes. (**a**) error map of surface 1; (**b**) etched region and masking layer of surface 1; (**c**) error map of surface 2; (**d**) etched region and masking layer of surface 2.

After *n* iterations, the residual error of the initial surface *R*_0_′(*x*,*y*) could be expressed as *RMS_surfn_* through the following optimization algorithm for MRF:

$$\mathrm{Minimize}\;T=\sum_{i=1}^{i=n}t_{i}$$

$$\mathrm{Subject}\;\mathrm{to}\;RMS_{surf_{n}}=\mathrm{nanstd}\left[R_{0}{}^{\prime }\left(x,y\right)-R_{n}\left(x,y\right)\right]\leq Q_{limit}$$

where *T* is the total etching time, and *Q*_limit_ is the threshold of *RMS_surfn_*. Figure 2 shows a schematic diagram to illustrate the etched region *EReg_i_*(*x*,*y*) and the masking layer region *MReg_i_*(*x*,*y*) on the surface during any one iteration of two mirrors.

Before the actual figuring, we needed to obtain the etching depth of RIE in response to the etching time by conducting many process experiments, so as to achieve the control of etching depth in each processing cycle. This is similar to the calculation and acquisition of dwell time in other polishing methods. In addition, unlike our previous attempts to perform reactive ion etching for fast figure-correction of transmission wave-front error on lightweight thin film diffraction elements and a quartz sub-mirror [25,30], an inkjet printer was used to print the UV-curable ink masking layers on the non-etching area *MReg_i_*(*x*,*y*) to protect the surface material. During each figuring cycle, when etching was completed, the masking layers were stripped using hydrogen peroxide, alcohol, and acetone, and the mirror was cleaned with deionized water. In order to improve figuring efficiency and reduce processing time, the etching depth and *MRF_i_* needed to be planned in each etching iteration according to the desired residual error requirements and the experimental equipment conditions. The figure correction of the quartz sub-mirror could be achieved gradually in combination with the above optimization algorithm for *MRF_i_*. Unlike capacitively coupled atmospheric pressure plasma processing [31], the material removal efficiency could vary depending on the size of the surface area of the etched workpiece. Furthermore, this plasma figure correction method had the advantage of not generating surface/sub-surface damage due to its low-temperature and non-contact processing characteristics, while the processing time was not influenced by the sample aperture, which enabled efficient and high-precision manufacturing of optical components.

## 3. Experimental

**Figure 3:**
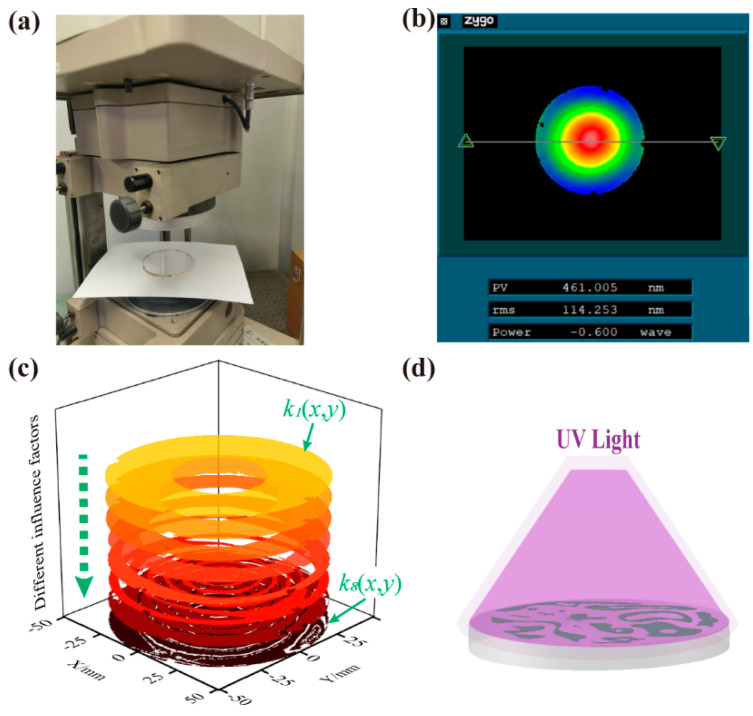
(**a**) Zygo GPI150 interferometer; (**b**) initial form error; (**c**) the influence factors used for the first eight distributed material removal functions; (**d**) schematic diagram of a printed UV-curable ink masking layer.

As shown in Figure 3a, the form error of the workpiece was measured using an interferometer (GPI150 by Zygo, Middlefield, CT, USA). The mirror was etched in a custom 650 mm aperture capacitively coupled plasma reactor (Beijing Jinsheng Weina Technology Co., Ltd., Beijing, China). The masking layers were printed onto the surface of the element by a UV inkjet printer (Shenzhen Ruifengcai Technology Co., Ltd., Shenzhen, China) with a stroke of 600 × 900 × 200 mm in the XYZ direction. To verify the effectiveness of figure correction, figuring experiments were conducted on a Corning 7980 planar quartz mirror with a diameter of 101.6 mm and a thickness of 5.5 mm. The detailed experimental conditions are listed in Table 1.

**Figure 4:**
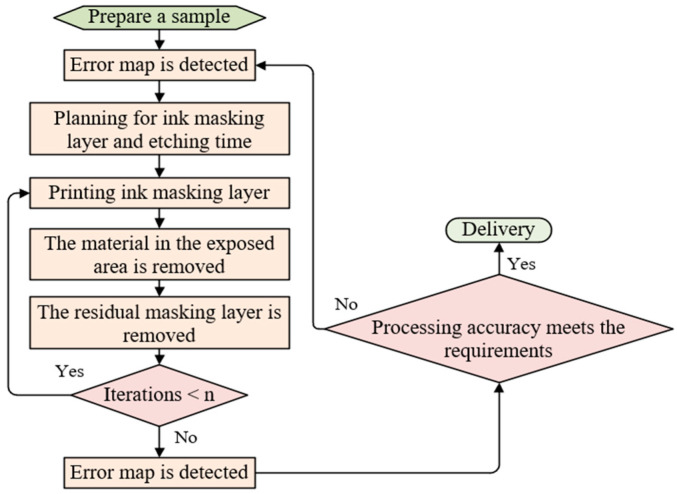
Process flow of the plasma figure correction method based on multiple distributed material removal functions.

Figure 3b shows the initial form error of the measured quartz mirror, and Figure 3d is the schematic diagram of a UV-curable ink masking layer printed on the surface of a preprocessed workpiece. The influence factors used for the first eight distributed material removal functions are shown from top to bottom in Figure 3c. As can be seen from the pattern of *k_i_*(*x*,*y*), the influence factors become more fragmented and scattered as the etching cycle increases, which is due to the gradual convergence of the residual error of the mirror. The process flow of the plasma figure correction method based on multiple distributed material removal functions is shown in Figure 4.

**Figure 5:**
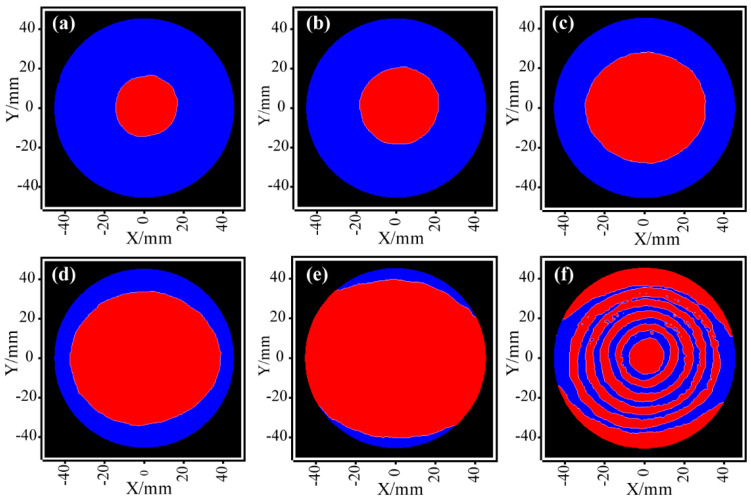
Material removal distributions for multiple distributed *MRF_i_*; (**a**–**f**) show the results in the first to sixth etching cycles, respectively.

Figure 5a–f show the distributions of the material removed by etching on the workpiece surface during the first six etching cycles. The blue part indicates *MReg_i_*(*x*,*y*) under the protection of the masking layers, whose material removal was 0, and the red area indicates *EReg_i_*(*x*,*y*), whose specific etching depth was determined by the corresponding planning algorithm. As shown in Figure 5, multiple distributed material removal functions could be delineated by printing a masking layer to obtain the desired material removal distribution in each figuring cycle. In contrast to the complex and tedious photolithography process, the inkjet printing method is less expensive, does not require special masks, and can flexibly create *MRF_i_* of any shape in the full-aperture range. Moreover, since RIE involves dry etching with anisotropic properties, the shape of the *MRF_i_* is no longer the usual Gaussian-like, but has nearly parallel removal properties to the material, which is not the same as some conventional polishing methods. Obviously, the use of multiple sets of different *MRF_i_* allows to obtain the desired material removal distribution and to remove the workpiece material in the full-aperture range.

## 4. Results and Discussion

**Figure 6:**
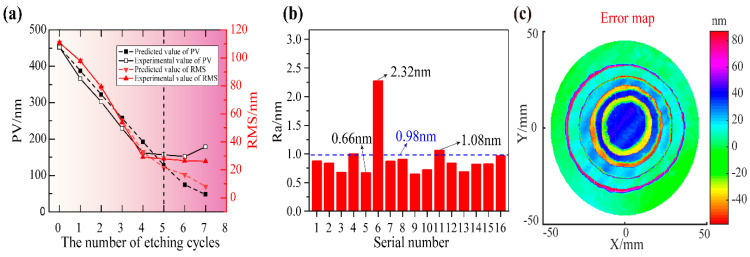
The result of the plasma figure correction: (**a**) results of the simulation and experiments; (**b**) surface roughness of the quartz sub-mirror before and after being etched; (**c**) error map between simulation result and experimental result.

Figure 6a,b shows the variation of the PV and RMS of the residual error with the etching cycle and the roughness of the etched workpiece surface, respectively. It can be seen that the simulated PV and RMS were basically the same as the detected values in the first five etching cycles, and the residual error converged gradually as the number of iterations increased. However, in the following two cycles, the detected PV and RMS did not continue to decrease, and the PV even appeared to increase. In addition, as shown in Figure 6b, 16 areas were selected equally spaced on the workpiece surface to measure the roughness of the quartz surface before and after being etched. Roughness (620 µm × 475 µm range) was measured by a 3D optical surface profiler (NewView 7300 by Zygo, Middlefield, CT, USA). Before being etched, the average roughness value of 16 areas on the surface was 0.98 nm. Most of the roughness on the workpiece surface after figuring was in the range of 0.66~1.08 nm. Therefore, the proposed plasma figure correction method does not distinctly deteriorate the roughness. Finally, the reflected wave-front error of the quartz sub-mirror converged from ~461 nm PV and ~114 nm RMS to ~157 nm PV and ~28 nm RMS through six etching iterations in total effective figuring time of ~47 min. The figure correction of the quartz sub-mirror based on multiple distributed material removal functions was achieved. 

Furthermore, the error map between the simulation result and the measurement result after the fourth iteration cycle is shown in Figure 6c. It can be clearly seen that there were some significant errors at the edges of each *MRF_i_*. In addition, the accumulation of the etching non-uniformity caused by multiple etches also produced a large error in the center area. Obviously, the alignment error between each masking layer and the manufacturing error of the masking layer will have a non-negligible influence on the etching results, which leads to the under-etching in the etched area or the over-etching in the mask layer area. Nevertheless, higher alignment and manufacturing precision of the masking layer can further improve figuring accuracy, such as choosing an inkjet printer with higher positioning accuracy and finer line widths, using laser radiography to manufacture the masking layers, or optimizing the alignment methods. In addition, in our earlier experiments, the etching non-uniformity measured by this experimental apparatus was less than ±6% [32], which has the potential to be further improved. Therefore, for higher precision plasma processing, the influence of the etching non-uniformity cannot be ignored to some extent.

**Figure 7:**
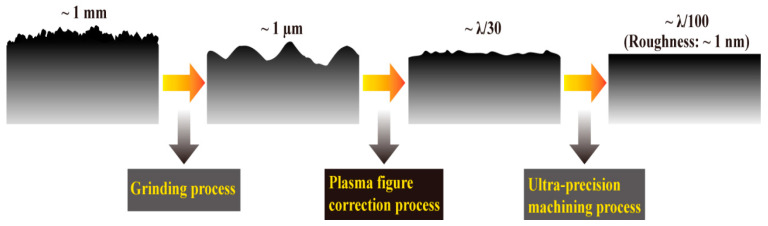
The role of plasma figure correction in the optical process chain.

Typically, optical components have approximately micron-level machining errors and millimeter-level subsurface damage layers after being milled and roughly polished. In order to take full advantage of the fast convergence of surface accuracy in the full-aperture range, the proposed plasma figure correction method based on multiple distributed material removal functions is executed between the grinding process (milling or rough polishing) and ultra-precision machining process, as shown in Figure 7. This processing method can provide gentle, non-contact, and parallel material removal in the whole-aperture range. It can be expected that the proposed plasma figure correction method will play an important role in the high-efficiency and low-damage processing technology of large-aperture optical components.

## 5. Conclusions

It is technically feasible to use the plasma figure correction method based on multiple distributed material removal functions. In each iterative process, the material removal function and its influence factor were calculated according to the form error of the component. The predicted residual errors were consistent with the experimental results. The material removal function of plasma processing could be cropped by the UV-curable ink masking layers in the full-aperture range, and the proposed plasma figure correction method was capable of fast figuring-correction of a quartz sub-mirror. It demonstrated the excellent performance and development potential of the method for figuring optical components such as quartz, silicon carbide, and even ultra-lightweight mirrors. Improving the manufacturing accuracy of the masking layers, updating the alignment method, optimizing the etching planning algorithm, and improving etching uniformity will be our main research goals in the future.

## Figures and Tables

**Table 1:** Detailed etching conditions.

**Reaction** **Gas**	CHF_3_, O_2_; the gas flow rate is 50 sccm and 95 sccm, respectively
**Chamber pressure**	1.5 Pa
**Distance between electrodes**	75 mm
**Masking layer material**	UV-curable ink
**Workpieces**	Plat mirror with 101.6 mm aperture
**Workpiece material**	SiO_2_
**Radio frequency power**	800 W
**Driving frequency**	13.56 MHz

## Data Availability

The data are available within the article.
